# Accuracy and Feasibility of Point-Of-Care White Blood Cell Count and C-Reactive Protein Measurements at the Pediatric Emergency Department

**DOI:** 10.1371/journal.pone.0129920

**Published:** 2015-06-02

**Authors:** Lauri Ivaska, Jussi Niemelä, Pia Leino, Jussi Mertsola, Ville Peltola

**Affiliations:** 1 Department of Paediatrics and Adolescent Medicine, Turku University Hospital, Turku, Finland; 2 Department of Paediatrics and Adolescent Medicine, University of Turku, Turku, Finland; 3 MediCity Research Laboratory, University of Turku, Turku, Finland; 4 Tykslab, Turku University Hospital, Turku, Finland; IIBB-CSIC-IDIBAPS, SPAIN

## Abstract

**Background:**

Several point-of-care (POC) tests are available for evaluation of febrile patients, but the data about their performance in acute care setting is sparse. We investigated the analytical accuracy and feasibility of POC tests for white blood cell (WBC) count and C-reactive protein (CRP) at the pediatric emergency department (ED).

**Methods:**

In the first part of the study, HemoCue WBC and Afinion AS100 CRP POC analyzers were compared with laboratory’s routine WBC (Sysmex XE-2100) and CRP (Modular P) analyzers in the hospital central laboratory in 77 and 48 clinical blood samples, respectively. The POC tests were then adopted in use at the pediatric ED. In the second part of the study, we compared WBC and CRP levels measured by POC and routine methods during 171 ED patient visits by 168 febrile children and adolescents. Attending physicians performed POC tests in capillary fingerprick samples.

**Results:**

In parallel measurements in the laboratory both WBC and CRP POC analyzers showed good agreement with the reference methods. In febrile children at the emergency department (median age 2.4 years), physician performed POC determinations in capillary blood gave comparable results with those in venous blood analyzed in the laboratory. The mean difference between POC and reference test result was 1.1 E9/L (95% limits of agreement from -6.5 to 8.8 E9/L) for WBC and -1.2 mg/L (95% limits of agreement from -29.6 to 27.2 mg/L) for CRP.

**Conclusions:**

POC tests are feasible and relatively accurate methods to assess CRP level and WBC count among febrile children at the ED.

## Introduction

Emergency departments (ED) are often crowded and operate on limited resources [1–4]. They meet patients with a wide range of disorders varying from common cold to severe conditions needing immediate care. Therefore, in the ED, aiming rapidly to preliminary diagnosis is of uppermost importance in managing triage and patient flow. Nevertheless, rapid clinical and laboratory evaluations should maintain high accuracy.

Point-of-care (POC) tests may provide several advantages for patient care in the ED setting. POC testing has been reported to facilitate pediatric patient flow and decrease the length of stay of children at the ED [5]. In addition to that, it can be cost-beneficial [6]. POC tests may contribute to patient triage and management in both up-to-date ED environments [7] and in resource-limited settings where no central laboratory is available [8]. Furthermore, the need for POC testing within patient isolation facilities, in order to avoid transport of potentially infectious samples, has emerged during the 2014 Ebola virus disease epidemic [9].

Biomarkers such as white blood cell (WBC) count, neutrophil cell count, C-reactive protein (CRP), procalcitonin and various cytokines have been suggested to be useful for quantifying the magnitude of inflammation or differentiating between bacterial and viral infection [10–14]. WBC and CRP are probably the most commonly used markers in the EDs and outpatient clinics. CRP POC tests are commercially available from several manufacturers and are widely used, and a POC device for rapid measurement of WBCs from capillary blood was introduced in 2000s.

Despite the potential usefulness of accurate and rapid detection tests for inflammatory markers, there is still only sparse data about performance of POC tests in clinical settings [15–16]. Among pediatric patients only few studies have evaluated the accuracy of POC CRP testing in acute care environment [17–19], and to our knowledge, there is only one clinical study focusing on POC WBC testing in children [20]. In this study we evaluated the analytical accuracy and practical feasibility of POC WBC and CRP tests in the pediatric ED setting. We also demonstrated the significance of evaluating POC tests actually at the site of care, in comparison with laboratory conditions.

## Materials and Methods

### Study conduct

In the first part of the study, the HemoCue WBC (HemoCue AB, Ängelholm, Sweden) and Afinion AS100 CRP (Axis-Shield PoC AS, Oslo, Norway) POC analyzers were compared with the standard laboratory WBC (Sysmex XE-2100 analyzer; Sysmex, Kobe, Japan) and CRP (Modular P, Roche Diagnostics, Mannheim, Germany) analyzers in the central laboratory of Turku University Hospital, Turku, Finland. Laboratory technicians performed POC WBC and CRP tests in parallel with the laboratory analyzer measurements in blood samples taken from pediatric and adult patients for other analytic purposes.

EDTA blood samples were divided for WBC measurements by Hemocue POC test and Sysmex analyzer. All WBC POC measurements were done twice from each sample and an average value was used in comparisons. Seventy-seven samples were analyzed for WBC. Twenty-four of these samples were micro-volume blood samples from neonates and 53 were standard venous samples from children or adults. Plasma separated from blood was used for CRP measurements both by Afinion AS100 POC test and Modular analyzer. Forty-eight samples were analyzed for CRP.

In the second part of the study, WBC and CRP POC tests were first incorporated in the routine practice at the pediatric ED, and then the POC test results were compared with measurements done in the central laboratory. Patients having WBC and CRP levels measured by both methods were identified from the hospital patient files, and were included in the study. The study was conducted during two periods (from May 2008 to January 2010 and from November 2011 to July 2013) at the Pediatric Emergency Department of Turku University Hospital. This pediatric ED serves a population of 70 000 children or adolescents. Most patients are referred to the ED from primary care clinics and a minority of patients comes directly from home. Study participants were 168 children and adolescents 0 to 16 years of age with 171 acute febrile infection episodes. The attending physician first examined each patient and then used POC testing according the routine practice, when clinically justified. After the POC determinations, venous blood was drawn for laboratory measurements of WBC count in the blood and CRP concentration in plasma from patients whom it was considered clinically necessary. A maximum delay of 4 hours between sampling for POC and laboratory analysis was allowed. User feedback was collected by discussions with ED physicians and nurses, who observed also patient and guardian opinions.

### Ethics statement

The study protocol was approved by the Institutional Review Board at the Clinical Research Center of The Turku University Hospital with a waiver of review by the Ethics Committee. For the purpose of this study, no extra samples were collected, and no changes to routine care of the patients were made. All data was collected retrospectively. For these reasons, no separate approval from the Ethics Committee was needed, and no informed consent were collected from the guardians. Authors were involved in the medical care of the patients and were not anonymized prior to statistical analysis.

### Training of personnel for performing POC tests

The staff physicians in our pediatric emergency department consist of registrars and consultants of pediatrics. All ED personnel operating the HemoCue WBC analyzer and CRP Afinion AS100 Analyzer (51 physicians) were trained according to manufacturer’s instructions and local practical guidelines. All users first underwent 30 minutes of theoretical training organized by the hospital central laboratory. Next, the users had to perform practical skills test of using the WBC and CRP POC analyzers, monitored by an authorized supervisor from the laboratory. All training together required less than 1 hour.

### CRP and WBC measurements

In the study conducted at the ED, the POC WBC and CRP analyses were performed in a patient examination room. A capillary blood drop needed for the analysis was collected by pricking the fingertip, or heel of infants less than 3 months of age, using a standard protocol as previously described [21]. Samples were collected and processed, and the results were interpreted by attending physicians during the first contact with the patient. WBC rapid test was performed with HemoCue analyzer and CRP was measured using Afinion AS100 analyzer according to the manufacturer's instructions. Venous blood samples were processed and analyzed in the hospital central laboratory by a Sysmex XE-2100 analyzer for WBC and Modular P analyzer for plasma CRP.

In brief, HemoCue WBC analyzer is a point-of-care testing system for the quantitative determination of WBC count based on microcuvette technology. Analysis can be done from a single blood drop (10 mL) collected with a glass cuvette by pricking the finger tip or alternatively from a venous EDTA blood sample. Results are obtained within 3 minutes. The measuring range of WBC count is 0.3–30.0 E9/L.

Afinion AS100 CRP is a point-of-care testing system based on an immunometric membrane flow-through assay for the quantitative determination of CRP either in capillary blood, serum or anti-coagulated venous blood (EDTA or heparin). Sample volume needed for each analysis is 1.5 μL. Results are obtained within 4 minutes and measuring range extends from 5 to 160 mg/L in serum/plasma and from 8 to 200 mg/L in whole blood.

### Statistical analyses

The agreement between POC and reference method results for WBC counts and CRP levels was analyzed according to the Bland-Altman method [22]. Two-way mixed model intraclass correlation coefficients were determined and descriptive data was analyzed using IBM SPSS Statistics, Version 21.0 (IBM Corp., Armonk, NY, USA). CRP results below the POC analyzer’s measuring range (<5 mg/L in plasma and <8 mg/L in whole blood) were referred as 2.5mg/L and 4 mg/L, respectively, in the statistical comparison. Results above the upper detection limit of either of the POC test system were excluded from the statistical analyses.

## Results

### Analytical accuracy of POC WBC and CRP tests

First, we evaluated the accuracy of HemoCue WBC and Afinion AS100 CRP POC analyzers compared with the Sysmex XE-2100 WBC and Modular P CRP analyzers in the laboratory. In the comparison between POC and reference WBC analyzers, a sampling method dependent difference was noticed. The mean difference between POC WBC value and reference WBC value was larger in micro-volume samples (-13%) than in standard venous samples (-1%) (S1 Table). Additional investigations revealed that the two micro-volume samples with the largest deviations (-82% and -93%) contained non-hemolyzed erythrocytes with Howell-Jolly bodies. When both micro-volume and venous samples (n = 77) were included, the mean difference between POC and reference WBC values was -5% (95% limits of agreement from -36% to 27%). The mean difference between POC and reference method CRP levels was 4% (95% limits of agreement from -16% to 25%) (S2 Table). The range of reference method results included in these comparisons was 1.9–31.5 E9/L for WBC count and 5–159 mg/L for plasma CRP concentration.

**Fig 1:**
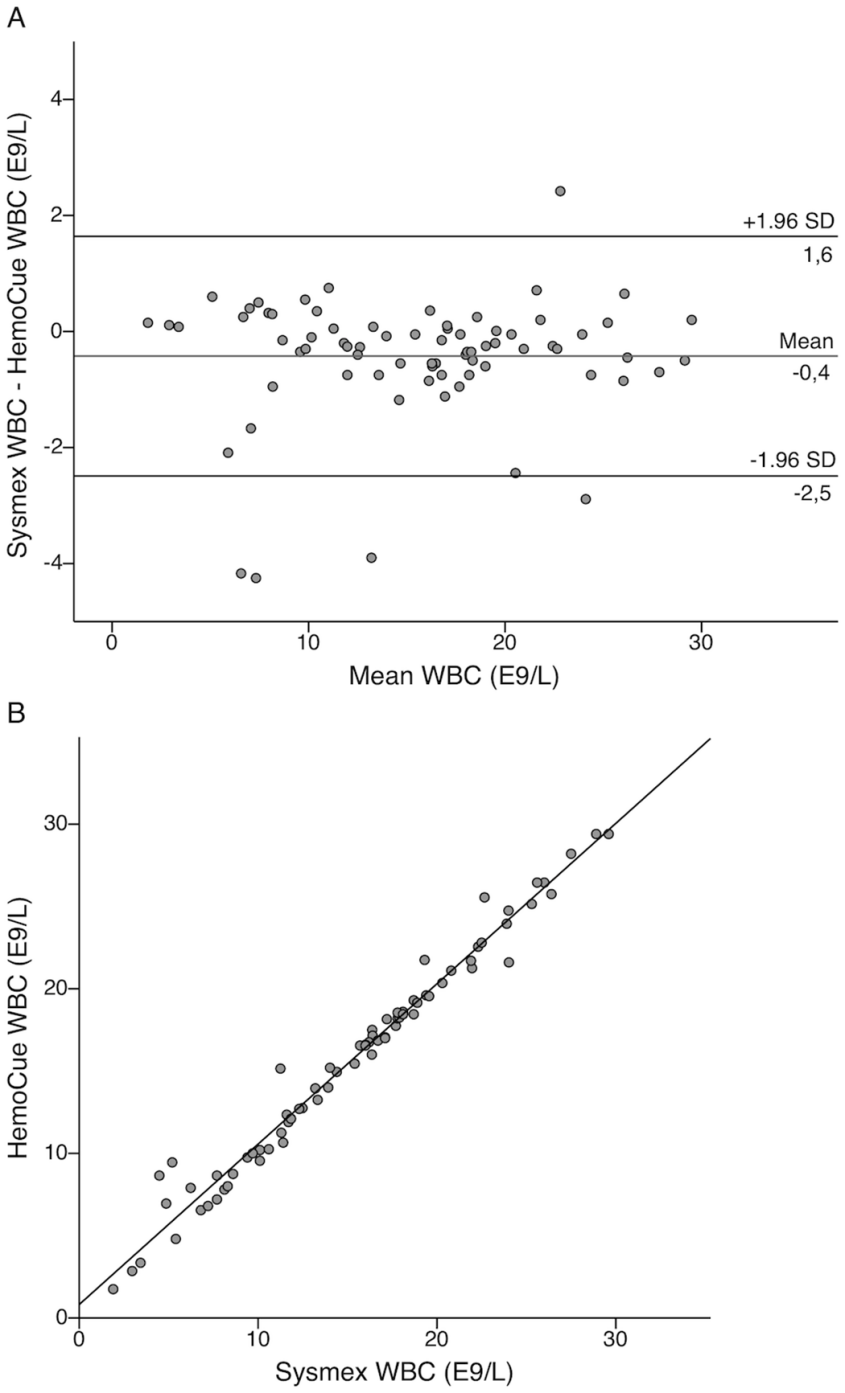
Bland-Altman and linear correlation plots for comparison of WBC methods in the laboratory. Bland-Altman plot presenting level of agreement (A) and a scatterplot showing linear correlation (B) between the HemoCue white blood cell (WBC) point-of-care test and Sysmex WBC results in 77 clinical blood samples. All testing was performed in the hospital’s central laboratory.

**Fig 2:**
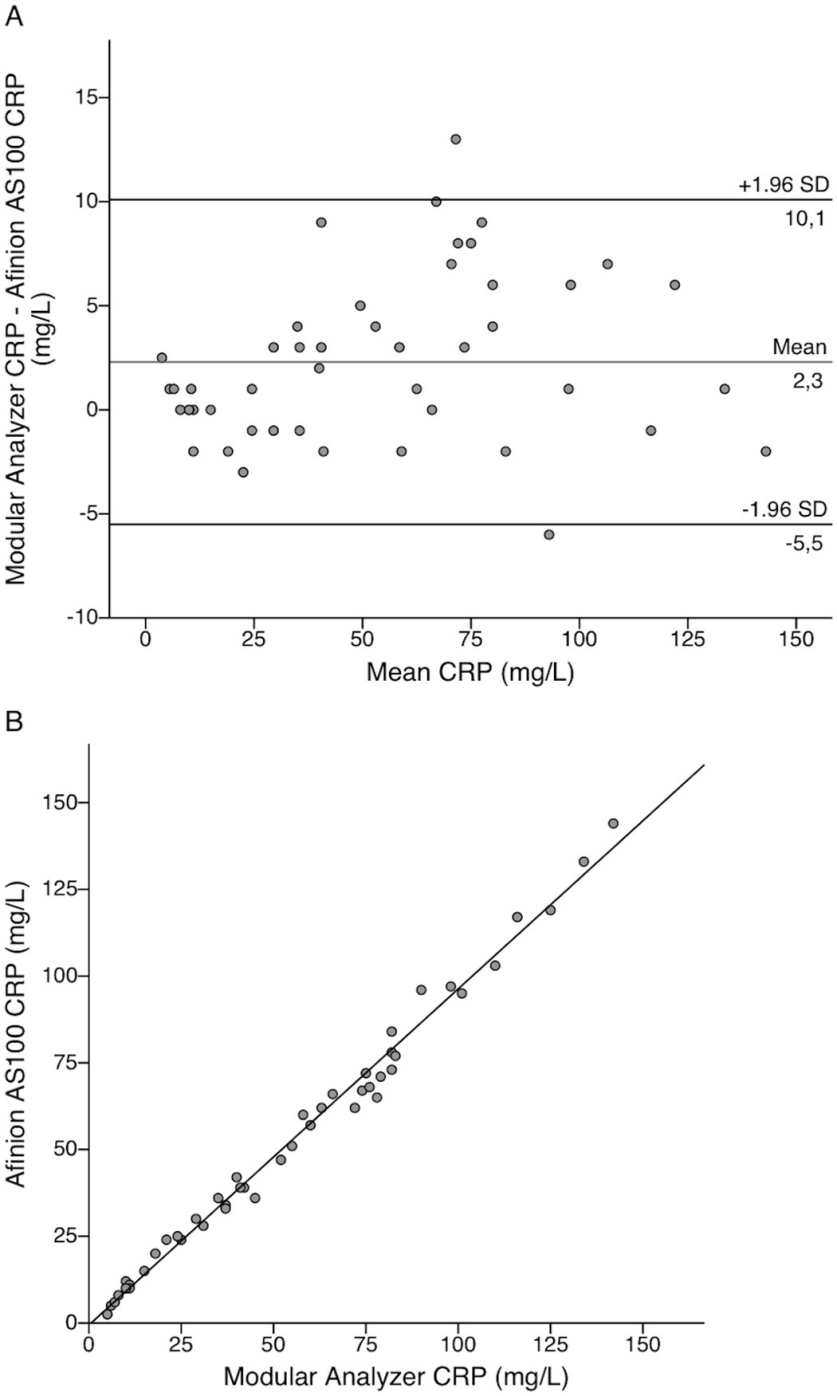
Bland-Altman and linear correlation plots for comparison of CRP methods in the laboratory. Bland-Altman plot presenting level of agreement (A) and a scatterplot showing linear correlation (B) between the Afinion AS100 C-reactive protein (CRP) point-of-care test and Modular CRP results in 48 clinical samples. All testing was performed in plasma samples in the hospital’s central laboratory.

Expressing the Bland-Altman agreements in numerical values, the mean difference between POC and reference method WBC count was -0.4 E9/L with 95% limits of agreement from -2.5 to 1.6 E9/L (Fig 1). Fig 1B presents the respective linear correlation for WBC. The mean difference between POC and reference method CRP was 2.3 mg/L with 95% limits of agreement from -5.5 to 10.1 mg/L (Fig 2). Single measure intraclass correlations between the POC and reference methods were 0.988 (95% CI 0.980–0.992) for WBC and 0.994 (95% CI 0.990–0.997) for CRP.

### Accuracy of POC WBC and CRP measurements in the ED

After implementation of POC WBC and CRP tests into routine practice at the pediatric ED, we tested the agreement between physician made POC tests and the reference laboratory analysis in samples collected during 171 visits by 168 febrile children. Patient characteristics are listed in Table 1. The median age of patients was 2.4 years (range, 0.04–16.38 years), and the majority of them were admitted to hospital after the ED visit.

**Table 1:** Characteristics of 168 children included in the study at the pediatric emergency department.

Age	No. Children	%
0 to <6 mo	28	16
6 mo to <1 yr	16	9
1 yr to < 3 yr	52	30
3 yr to < 7 yr	40	23
7–17 yr	35	20
**Sex**		
Male	78	46
Female	93	54
**Diagnosis** ^1^		
Bacteremia^2^	11	6
Pneumonia	40	23
Pyelonephritis	28	16
Osteoarticular infections	2	1
Acute abdomen	16	9
Local bacterial^3^	28	16
Viral/FWO	42	25
Inflammatory	2	1
Hyperglycemia	2	1
**Hospital admittance**		
Yes	149	87
No	22	13

^1^As registered in the medical patient files.

^2^Both, documented or suspected bacteremia.

^3^Acute otitis media, bacterial tonsillitis, abscess.

**Fig 3:**
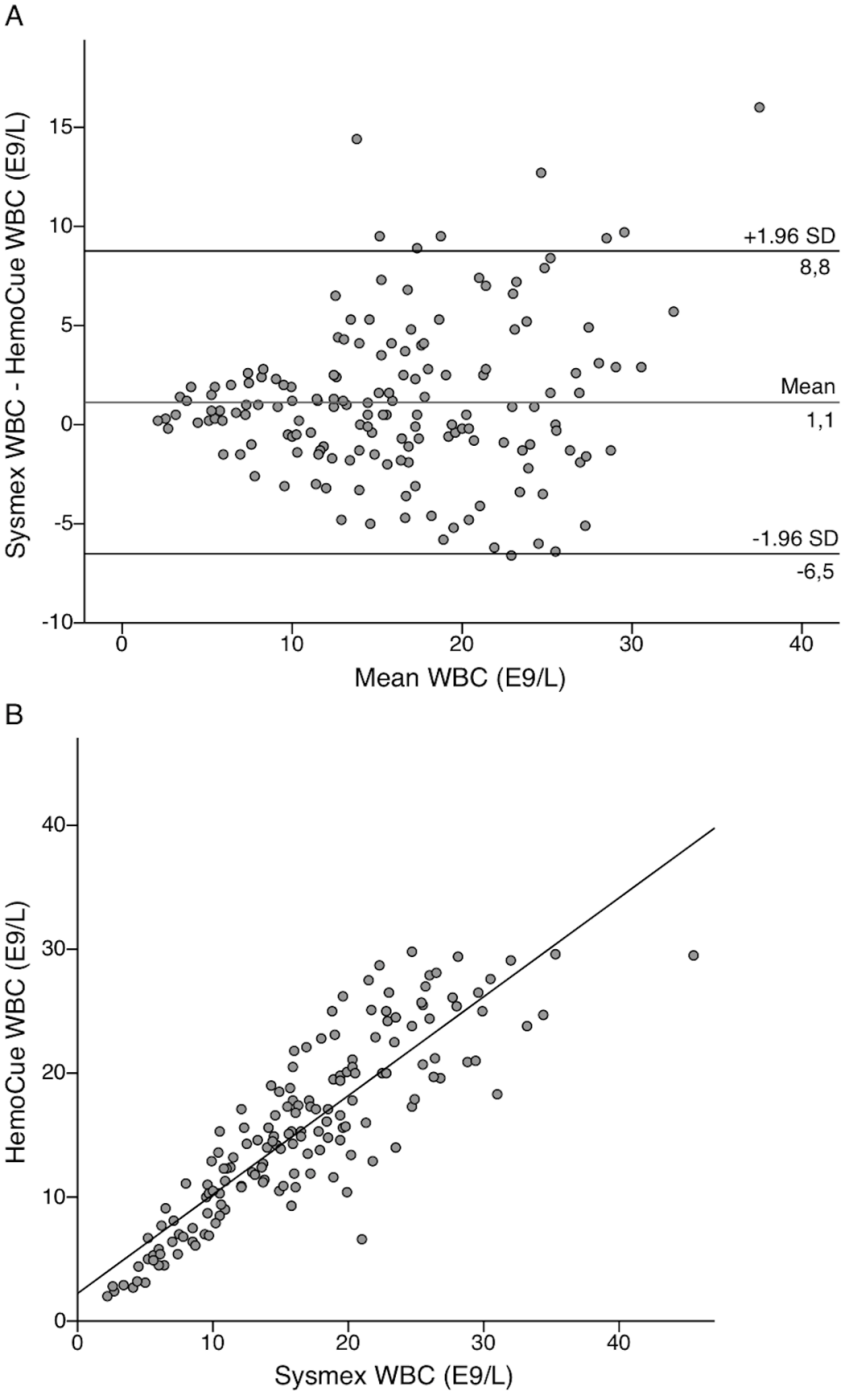
Bland-Altman and linear correlation plots for comparison of WBC methods in the emergency department. Bland-Altman plot presenting level of agreement (A) and a scatterplot showing linear correlation (B) between the HemoCue white blood cell (WBC) point-of-care (POC) test and Sysmex WBC results in 171 pediatric emergency department (ED) visits. Clinicians performed POC testing at the ED in capillary blood samples and Sysmex analysis was done in venous blood samples in the laboratory.

**Fig 4:**
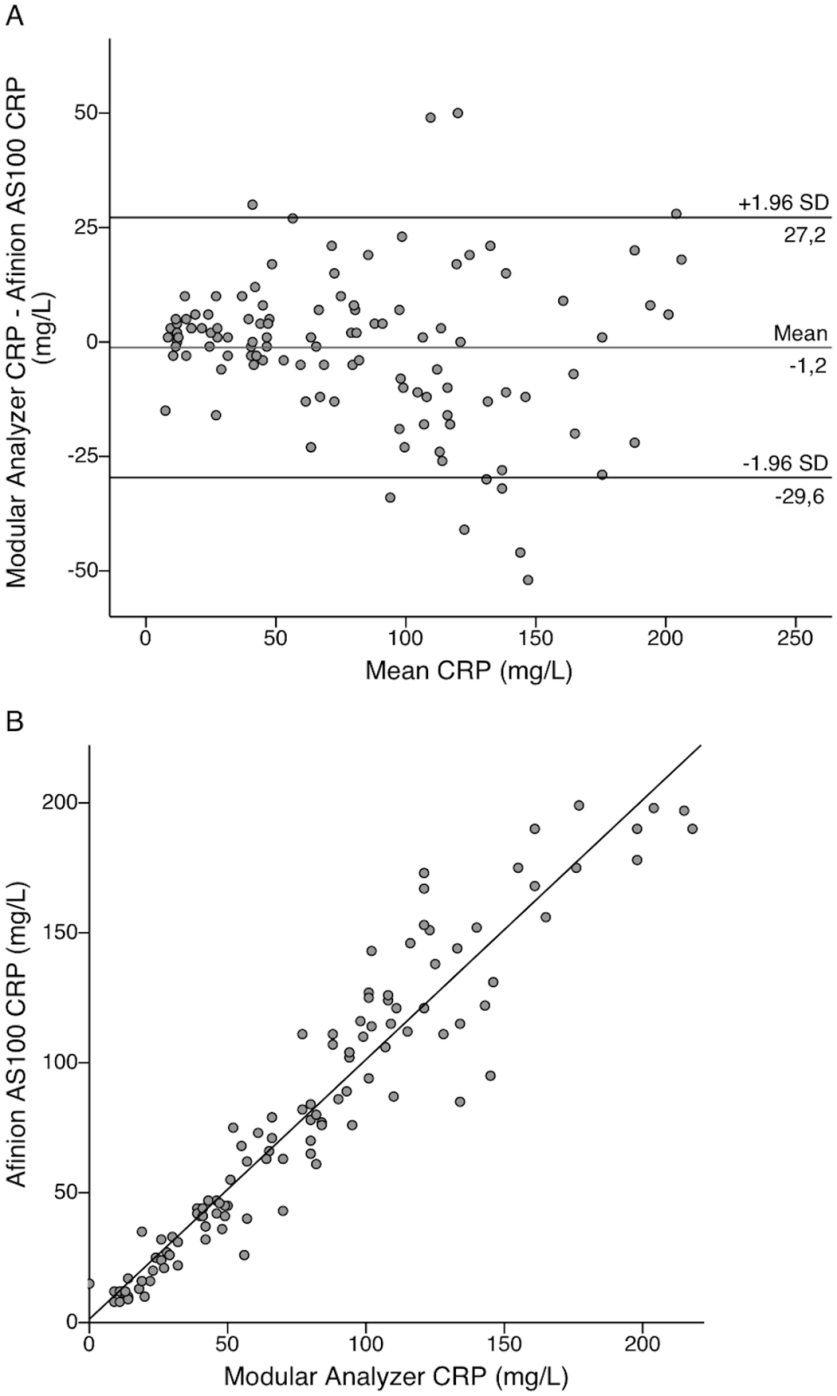
Bland-Altman and linear correlation plots for comparison of CRP methods in the emergency department. Bland-Altman plot presenting level of agreement (A) and a scatterplot showing linear correlation (B) between the Afinion AS100 C-reactive protein (CRP) point-of-care (POC) test and Modular CRP results in 171 pediatric emergency department (ED) visits. Clinicians performed POC testing at the ED in capillary blood samples and Modular CRP analysis was done in plasma separated from venous blood samples in the laboratory.

The mean difference between POC and reference test WBC count was 1.1 E9/L with 95% limits of agreement from -6.5 to 8.8 E9/L (Fig 3). Fig 3B presents the linear correlation for WBC within this patient population. The mean difference between POC and reference test CRP was -1.2 mg/L with 95% limits of agreement from -29.6 to 27.2 mg/L (Fig 4) (see also S3 Table). The single measure intraclass correlations between the POC and reference methods were 0.864 (95% CI 0.818–0.899) for WBC count and 0.967 (95% CI 0.955–0.976) for CRP.

In patients with POC WBC levels exceeding the upper limit of measuring range (>30 E9/L; n = 14), respective WBC values varied from 22.1 to 53.5 E9/L (mean 33.3 E9/L) by the reference method. The CRP values for patients with POC test result exceeding the upper limit of range (>200 mg/L; n = 24) were 163–409 mg/L (mean 242 mg/L) by the reference method. In children with POC CRP below the range (<8 mg/L; n = 34), the reference method levels varied from 0 to 16 mg/L (mean 3.2 mg/L).

### Clinical experiences

For attending physician, POC testing in the pediatric ED required a short (approximately 1 minute) hands on time for sampling and 3 and 4 minutes for processing with HemoCue WBC and Afinion CRP, respectively. Based on our observations, in the pediatric ED setting, POC test results were consistently available within 5 minutes subsequent to sample collection. Technical errors in the POC analyses were rare. The feedback from the ED personnel, patients and their guardians on use of POC tests in the routine practice was generally positive, reflecting the rapid return of test results and proceeding of the patient care. Negative feedback included occasional errors in the Hemocue WBC testing, and the somewhat confusing error codes of Afinion CRP test, which both could be avoided by additional training in proper performance of the tests.

## Discussion

The main findings of this study are the good analytical accuracy of POC CRP and WBC measurements and the feasibility of using these tests in febrile patients presenting at the pediatric ED. Comparing the POC tests with the reference tests in the laboratory, we found a fairly good agreement for both WBC and CRP. When the POC tests were further evaluated in routine practice at the pediatric ED, larger deviations from laboratory results were seen, but still the accuracy of both tests was acceptable and the rapidity of analyses was considered a major advantage.

Good analytical accuracy is a prerequisite for a useful test system. We confirmed that the performance of POC WBC and CRP tests in comparison with the hospital routine laboratory methods was generally as good as expected by the information from the manufacturers. In this respect, two micro-volume samples made an exception. Howell-Jolly bodies (nuclear remnants of basophilic DNA in red blood cells) are occasionally seen in the peripheral blood of full-term, but especially of preterm neonates. This can be due to developmental splenic immaturity [23]. The POC WBC test system apparently misinterpreted these erythrocytes as leukocytes, leading to falsely elevated results. Conditions causing resistance against erythrocyte hemolysis have been recognized to possibly interfere the WBC count by the HemoCue analyzer [24]. However, preterm infants are a minority among ED patients, and therefore, analyzer’s utility in this setting is hardly affected.

The ED-performed POC testing was less precise than POC analysis done in the laboratory, in comparison with the reference methods. This may partly be due to the fact that the capillary samples for POC testing and venous blood for reference analysis were not always collected simultaneously at the ED, although within a 4-hour time range. WBC levels may alter faster, especially during pneumococcal infection, whereas CRP peaks in approximately 48 hours after onset of fever [25,26]. In addition, the sampling method (capillary vs. venous sampling) may have some effect on the results, although a report on the POC measurement of CD4 T cell count did not find significant differences depending on the sampling [27]. Furthermore, capillary sampling may be vulnerable to technical issues such as too hard squeezing of finger for prick sampling. All of our ED physicians (51 clinicians) performed sampling and POC testing, which makes operator dependent differences possible despite proper training of all test operators. However, the agreement between POC and laboratory methods for both WBC and CRP were considered sufficient for the purposes of identifying children at risk of severe bacterial infection.

User experiences were mainly positive. In accordance with studies evaluating POC tests in the ED or outpatient clinics [5,17,28], our personnel valued the rapid availability of test results as an important feature, being helpful in the clinical decision-making especially during crowded periods (e.g. virus epidemic seasons) and on call hours. Notably, capillary sampling of blood is faster, less invasive, less painful and needs fewer resources than venous blood sampling. Recently, the suitability of capillary fingerprick blood samples for accurate WBC differential count determination by a POC method was shown in a small sample of healthy individuals [21].

In this study we did not determine the effect of POC testing on patient flow or costs. However, a previous study by Hsiao et al. reported that POC testing facilitates pediatric patient flow and decreases the length of stay in ED settings [5]. Medical personnel reported similar experiences in our study. Short waiting times for test results were regarded important also for patient satisfaction.

Our study has some limitations due to its arrangement at the ED. The pragmatic composition of this part of the study (including finger prick sampling for POC tests vs. venous sampling for reference tests, and the time delay between POC and laboratory test sampling) should, however, be considered as a strength rather than a limitation, because thus we could demonstrate the effects on WBC and CRP results caused by incorporating POC tests in the routine practice. The different performance of POC tests at the actual "point-of-care" compared with laboratory environment highlights the importance of conducting POC method evaluations at the clinical settings and thus verifying the performance of the method in the hands of the final users of the tests.

## Conclusions

Our data presents the feasibility and sufficient accuracy of point-of-care white blood cell count and C-reactive protein measurements in the pediatric emergency department. This approach may benefit small outpatient clinics or resource poor settings, and it may contribute to managing patient flow efficiently at crowded emergency outpatient clinics of pediatric hospitals. Further studies are needed to evaluate point-of-care tests’ cost-benefit and impact on clinical decision-making.

## Supporting Information

S1 Table WBC count comparison in the laboratory. (DOC)

S2 Table CRP comparison in the laboratory. (DOC)

S3 Table POC comparisons (WBC and CRP) at the ED. (DOC)
