# Supplementary material for: Accuracy and Feasibility of Point-Of-Care White Blood Cell Count and C-Reactive Protein Measurements at the Pediatric Emergency Department
Source: PLoS One. 2015 Jun 2;10(6):e0129920. doi: 10.1371/journal.pone.0129920 (PMC4452774; doi:10.1371/journal.pone.0129920)
Supplement: S1 Table — (DOC) [file pone.0129920.s001.doc]

**Table S1:** WBC count comparison in the laboratory.

| **Test No** | **WBC POC (E9/L)** | **WBC LAB (E9/L)** |  |
| --- | --- | --- | --- |
| 1 | 21,25 | 21,96 |  |
| 2 | 17,5 | 16,38 |  |
| 3 | 24,75 | 24 |  |
| 4 | 1,75 | 1,9 |  |
| 5 | 23,95 | 23,9 |  |
| 6 | 28,2 | 27,5 |  |
| 7 | 10,2 | 10,1 |  |
| 8 | 12,35 | 11,6 |  |
| 9 | 9,75 | 9,4 |  |
| 10 | 20,35 | 20,3 |  |
| 11 | 2,85 | 2,96 |  |
| 12 | 15,15 | 11,25 | micro-volume sample |
| 13 | 7,8 | 8,12 |  |
| 14 | 11,9 | 11,7 |  |
| 15 | 10 | 9,7 |  |
| 16 | 6,55 | 6,8 |  |
| 17 | 11,25 | 11,3 |  |
| 18 | 14,95 | 14,4 |  |
| 19 | 13,95 | 13,2 |  |
| 20 | 18,15 | 17,2 | micro-volume sample |
| 21 | 25,55 | 22,66 | micro-volume sample |
| 22 | 13,25 | 13,33 | micro-volume sample |
| 23 | 7,2 | 7,7 | micro-volume sample |
| 24 | 15,2 | 14,02 | micro-volume sample |
| 25 | 17,05 | 17,1 |  |
| 26 | 26,45 | 26 |  |
| 27 | 29,4 | 29,6 |  |
| 28 | 8,65 | 7,7 | micro-volume sample |
| 29 | 10,65 | 11,4 | micro-volume sample |
| 30 | 21,75 | 19,31 | micro-volume sample |
| 31 | 6,95 | 4,86 | micro-volume sample |
| 32 | 10,25 | 10,6 | micro-volume sample |
| 33 | 25,15 | 25,3 |  |
| 34 | 26,45 | 25,6 |  |
| 35 | 17,15 | 16,4 |  |
| 36 | 9,45 | 5,2 | micro-volume sample |
| 37 | 4,8 | 5,4 | micro-volume sample |
| 38 | 8 | 8,3 | micro-volume sample |
| 39 | >30 | 31,3 |  |
| 40 | >30 | 28,9 |  |
| 41 | 16,75 | 16,2 |  |
| 42 | 22,55 | 22,3 |  |
| 43 | 16,6 | 16 |  |
| 44 | 29,4 | 28,9 |  |
| 45 | 9,55 | 10,1 | micro-volume sample |
| 46 | 8,65 | 4,48 | micro-volume sample |
| 47 | 12,75 | 12,48 | micro-volume sample |
| 48 | 18,45 | 18,7 |  |
| 49 | 17,75 | 17,7 |  |
| 50 | 22,8 | 22,5 |  |
| 51 | 19,6 | 19,4 |  |
| 52 | 16,85 | 16,7 |  |
| 53 | 12,1 | 11,84 | micro-volume sample |
| 54 | 14 | 13,92 | micro-volume sample |
| 55 | 12,7 | 12,3 | micro-volume sample |
| 56 | 21,1 | 20,8 |  |
| 57 | 18,6 | 18,1 |  |
| 58 | 25,75 | 26,4 |  |
| 59 | 19,3 | 18,7 |  |
| 60 | 16,55 | 15,7 |  |
| 61 | 21,6 | 24,02 | micro-volume sample |
| 62 | 15,45 | 15,4 |  |
| 63 | >30 | 31,47 | micro-volume sample |
| 64 | 7,9 | 6,23 | micro-volume sample |
| 65 | 16 | 16,36 | micro-volume sample |
| 66 | 19,15 | 18,9 |  |
| 67 | 18,2 | 17,8 |  |
| 68 | 18,25 | 17,9 |  |
| 69 | 18,55 | 17,8 |  |
| 70 | 21,7 | 21,9 |  |
| 71 | 17 | 17,1 |  |
| 72 | 16,55 | 16 |  |
| 73 | 19,55 | 19,56 |  |
| 74 | 18,45 | 18,1 |  |
| 75 | 6,8 | 7,2 |  |
| 76 | 8,75 | 8,6 |  |
| 77 | 3,35 | 3,43 |  |
