# Supplementary material for: Accuracy and Feasibility of Point-Of-Care White Blood Cell Count and C-Reactive Protein Measurements at the Pediatric Emergency Department
Source: PLoS One. 2015 Jun 2;10(6):e0129920. doi: 10.1371/journal.pone.0129920 (PMC4452774; doi:10.1371/journal.pone.0129920)
Supplement: S2 Table — (DOC) [file pone.0129920.s002.doc]

**Table S2:** CRP comparison in the laboratory.

| **Test No** | **CRP POC (mg/L)** | **CRP LAB (mg/L)** |
| --- | --- | --- |
| 1 | 12 | 10 |
| 2 | 11 | 11 |
| 3 | 20 | 18 |
| 4 | 24 | 25 |
| 5 | 36 | 35 |
| 6 | 39 | 42 |
| 7 | 51 | 55 |
| 8 | 66 | 66 |
| 9 | 67 | 74 |
| 10 | 84 | 82 |
| 11 | 24 | 21 |
| 12 | 30 | 29 |
| 13 | 57 | 60 |
| 14 | 28 | 31 |
| 15 | 62 | 72 |
| 16 | 73 | 82 |
| 17 | 60 | 58 |
| 18 | 62 | 63 |
| 19 | 15 | 15 |
| 20 | 34 | 37 |
| 21 | 47 | 52 |
| 22 | 36 | 45 |
| 23 | 10 | 11 |
| 24 | 103 | 110 |
| 25 | 117 | 116 |
| 26 | 39 | 41 |
| 27 | 68 | 76 |
| 28 | 95 | 101 |
| 29 | 97 | 98 |
| 30 | 96 | 90 |
| 31 | 25 | 24 |
| 32 | <5 | 5 |
| 33 | 5 | 6 |
| 34 | 6 | 7 |
| 35 | 8 | 8 |
| 36 | 10 | 10 |
| 37 | 10 | 10 |
| 38 | 72 | 75 |
| 39 | 119 | 125 |
| 40 | 133 | 134 |
| 41 | 144 | 142 |
| 42 | >160 | 159 |
| 43 | 78 | 82 |
| 44 | 71 | 79 |
| 45 | 42 | 40 |
| 46 | 77 | 83 |
| 47 | 65 | 78 |
| 48 | 33 | 37 |
