# Supplementary material for: Accuracy and Feasibility of Point-Of-Care White Blood Cell Count and C-Reactive Protein Measurements at the Pediatric Emergency Department
Source: PLoS One. 2015 Jun 2;10(6):e0129920. doi: 10.1371/journal.pone.0129920 (PMC4452774; doi:10.1371/journal.pone.0129920)
Supplement: S3 Table — (DOC) [file pone.0129920.s003.doc]

**Table S3:** POC comparisons (WBC and CRP) at the ED.

| **ED visit No** | **WBC POC (E9/L)** | **WBC LAB (E9/L)** | **CRP POC (mg/L)** | **CRP LAB (mg/L)** |
| --- | --- | --- | --- | --- |
| 1 | 3,1 | 5 | 68 | 55 |
| 2 | 5 | 5,2 | <8 | 8 |
| 3 | 5,3 | 5,6 | <8 | 1 |
| 4 | 5,8 | 6 | <8 | 3 |
| 5 | 7,7 | 6,2 | 32 | 26 |
| 6 | 4,5 | 6,4 | <8 | 1 |
| 7 | 6,4 | 7 | >200 | 238 |
| 8 | 7 | 7,5 | >200 | 338 |
| 9 | 6,4 | 8,5 | 121 | 111 |
| 10 | 10 | 9,5 | 25 | 24 |
| 11 | 11 | 9,6 | 73 | 61 |
| 12 | 10,3 | 9,7 | <8 | 4 |
| 13 | 13,6 | 10,4 | 152 | 140 |
| 14 | 15,3 | 10,5 | 151 | 123 |
| 15 | 10,3 | 10,5 | >200 | 248 |
| 16 | 9 | 10,9 | 12 | 12 |
| 17 | 13,2 | 11,5 | 198 | 204 |
| 18 | 10,9 | 12,1 | 77 | 84 |
| 19 | 14,6 | 13,3 | 102 | 94 |
| 20 | 11,4 | 13,8 | 111 | 77 |
| 21 | 14,9 | 14,5 | >200 | 277 |
| 22 | 14,2 | 14,7 | 175 | 176 |
| 23 | 18,5 | 14,9 | >200 | 216 |
| 24 | 13,9 | 15 | <8 | 3 |
| 25 | 18,8 | 15,7 | >200 | 236 |
| 26 | 15,3 | 15,8 | 44 | 39 |
| 27 | 17,8 | 15,9 | 124 | 108 |
| 28 | 20,5 | 15,9 | 115 | 109 |
| 29 | 14,3 | 15,9 | <8 | 1 |
| 30 | 10,8 | 16,1 | 41 | 40 |
| 31 | 17,4 | 16,3 | 94 | 101 |
| 32 | 15,3 | 16,5 | 82 | 77 |
| 33 | 13,5 | 17 | 190 | 218 |
| 34 | 17,8 | 17,1 | 114 | 102 |
| 35 | 17,3 | 17,2 | <8 | 3 |
| 36 | 15,3 | 17,8 | >200 | 163 |
| 37 | 25 | 18,8 | 55 | 51 |
| 38 | 19,5 | 18,9 | 12 | 9 |
| 39 | 11,6 | 18,9 | 79 | 66 |
| 40 | 23,1 | 19 | >200 | 193 |
| 41 | 19,8 | 19,4 | >200 | 213 |
| 42 | 19,4 | 19,4 | 26 | 56 |
| 43 | 26,2 | 19,6 | 106 | 107 |
| 44 | 20,1 | 19,9 | 86 | 90 |
| 45 | 10,4 | 19,9 | >200 | 355 |
| 46 | 6,6 | 21 | <8 | 1 |
| 47 | 27,5 | 21,5 | 62 | 57 |
| 48 | 25,1 | 21,7 | 42 | 46 |
| 49 | >30 | 22,1 | 17 | 14 |
| 50 | 28,7 | 22,3 | 104 | 94 |
| 51 | 20 | 22,5 | 12 | 11 |
| 52 | 24,2 | 22,9 | >200 | 229 |
| 53 | >30 | 23,1 | 115 | 134 |
| 54 | 14 | 23,5 | 110 | 99 |
| 55 | 24,5 | 23,5 | 197 | 215 |
| 56 | 23,8 | 24,7 | 42 | 39 |
| 57 | 29,8 | 24,7 | 41 | 49 |
| 58 | 25,5 | 25,5 | 33 | 30 |
| 59 | 27 | 25,7 | 27 | 28 |
| 60 | 27,9 | 26 | 63 | 70 |
| 61 | 28,1 | 26,5 | 89 | 93 |
| 62 | 19,6 | 26,8 | 84 | 80 |
| 63 | >30 | 27,6 | 45 | 50 |
| 64 | 26,1 | 27,7 | 41 | 41 |
| 65 | 25,4 | 28 | <8 | 4 |
| 66 | 20,9 | 28,8 | 78 | 80 |
| 67 | >30 | 29 | 8 | 1 |
| 68 | >30 | 30,1 | 66 | 65 |
| 69 | 27,6 | 30,5 | >200 | 298 |
| 70 | >30 | 31,2 | 75 | 52 |
| 71 | >30 | 32 | 47 | 46 |
| 72 | >30 | 34 | 63 | 64 |
| 73 | >30 | 41,6 | 168 | 161 |
| 74 | >30 | 47 | 71 | 66 |
| 75 | >30 | 24,9 | >200 | 202 |
| 76 | 29,5 | 45,5 | 35 | 19 |
| 77 | 29,1 | 32 | 153 | 121 |
| 78 | 26,5 | 23 | 173 | 121 |
| 79 | 25 | 29,9 | 127 | 101 |
| 80 | 21,1 | 20,3 | >200 | 187 |
| 81 | 17,3 | 24,7 | >200 | 235 |
| 82 | 16,8 | 16,1 | >200 | 250 |
| 83 | 16 | 21,3 | >200 | 195 |
| 84 | 14,6 | 19,4 | >200 | 178 |
| 85 | 10,8 | 12,1 | >200 | 198 |
| 86 | 9,1 | 6,5 | >200 | 205 |
| 87 | 16,1 | 18,4 | 190 | 161 |
| 88 | 12,7 | 13,7 | 178 | 198 |
| 89 | 15,6 | 12,3 | 175 | 155 |
| 90 | 24,4 | 26 | 167 | 121 |
| 91 | 15,1 | 15,6 | 156 | 165 |
| 92 | 11,9 | 17,2 | 146 | 116 |
| 93 | 14 | 14 | 144 | 133 |
| 94 | 8,5 | 10,5 | 143 | 102 |
| 95 | 16,6 | 19,4 | 138 | 125 |
| 96 | 12,9 | 21,8 | 131 | 146 |
| 97 | 22,5 | 23,4 | 126 | 108 |
| 98 | 11,3 | 10,9 | 125 | 101 |
| 99 | 17,1 | 12,1 | 122 | 143 |
| 100 | 14,5 | 14,4 | 121 | 121 |
| 101 | 19 | 14,3 | 116 | 98 |
| 102 | 10,5 | 10 | 112 | 115 |
| 103 | 17,9 | 24,9 | 111 | 88 |
| 104 | 17,1 | 18,5 | 111 | 128 |
| 105 | 23,8 | 33,2 | 107 | 88 |
| 106 | 7,9 | 10,2 | 13 | 18 |
| 107 | 29,6 | 35,3 | 87 | 110 |
| 108 | 17,8 | 20,3 | <8 | 8 |
| 109 | 10,5 | 14,9 | 8 | 9 |
| 110 | 5,4 | 7,4 | <8 | 0 |
| 111 | 25,7 | 25,4 | 37 | 42 |
| 112 | 7,0 | 9,4 | <8 | 0 |
| 113 | 18,3 | 31,0 | 40 | 57 |
| 114 | 5,4 | 6,1 | <8 | 3 |
| 115 | 2,4 | 2,7 | <8 | 2 |
| 116 | 17,3 | 15,5 | 65 | 80 |
| 117 | 6,7 | 5,2 | <8 | 0 |
| 118 | 15,6 | 14,1 | 80 | 82 |
| 119 | 20,5 | 20,3 | 8 | 11 |
| 120 | 29,4 | 28,1 | 61 | 82 |
| 121 | 15,6 | 19,6 | 32 | 42 |
| 122 | 2,7 | 4,1 | <8 | 3 |
| 123 | 12,4 | 11,3 | <8 | 0 |
| 124 | >30 | 35,6 | 76 | 95 |
| 125 | 12,3 | 11,0 | <8 | 4 |
| 126 | 2,0 | 2,2 | <8 | 1 |
| 127 | >30 | 53,5 | 15 | 0 |
| 128 | 21,8 | 16,0 | 70 | 80 |
| 129 | 13,8 | 17,9 | 45 | 49 |
| 130 | 25,0 | 22,8 | 16 | 22 |
| 131 | 14,3 | 12,5 | 199 | 177 |
| 132 | 20,7 | 25,5 | 95 | 145 |
| 133 | 14,8 | 18,5 | 190 | 198 |
| 134 | 12,4 | 13,6 | <8 | 5 |
| 135 | 6,8 | 7,8 | <8 | 0 |
| 136 | 6,1 | 8,7 | <8 | 10 |
| 137 | 10,9 | 15,2 | 16 | 19 |
| 138 | 14,9 | 16,5 | <8 | 5 |
| 139 | 9,4 | 10,6 | 10 | 20 |
| 140 | 7,5 | 8,5 | <8 | 0 |
| 141 | 21,0 | 29,4 | 24 | 26 |
| 142 | 17,1 | 17,6 | 22 | 32 |
| 143 | 9,3 | 15,8 | 21 | 27 |
| 144 | 6,9 | 9,7 | <8 | 6 |
| 145 | 2,9 | 3,4 | <8 | 7 |
| 146 | 4,5 | 6,0 | 11 | 13 |
| 147 | 26,5 | 29,6 | 46 | 47 |
| 148 | 22,8 | 18,0 | 31 | 32 |
| 149 | 11,2 | 13,7 | <8 | 4 |
| 150 | 13,4 | 20,2 | 10 | 14 |
| 151 | 12,9 | 9,9 | <8 | 16 |
| 152 | 15,7 | 19,8 | 47 | 43 |
| 153 | 3,2 | 4,4 | 16 | 19 |
| 154 | 20,0 | 20,5 | >200 | 242 |
| 155 | 24,7 | 34,4 | 76 | 84 |
| 156 | 8,7 | 9,6 | 43 | 70 |
| 157 | 12,0 | 12,9 | 20 | 23 |
| 158 | 21,2 | 26,4 | >200 | 202 |
| 159 | 4,9 | 5,6 | 44 | 41 |
| 160 | 11,8 | 13,1 | <8 | 2 |
| 161 | 20,0 | 22,8 | 9 | 14 |
| 162 | 11,9 | 16,0 | 12 | 13 |
| 163 | 2,8 | 2,6 | <8 | 2 |
| 164 | 11,1 | 8,0 | <8 | 1 |
| 165 | 4,4 | 4,5 | <8 | 1 |
| 166 | 22,1 | 16,9 | >200 | 409 |
| 167 | >30 | 34,0 | >200 | 291 |
| 168 | 16,6 | 14,6 | <8 | 0 |
| 169 | 12,3 | 10,8 | 26 | 29 |
| 170 | 19,7 | 26,3 | 36 | 48 |
| 171 | 22,9 | 22,0 | 85 | 134 |
